# Negative impact of striae gravidarum in maternal mental health^☆^

**DOI:** 10.1016/j.abd.2024.09.012

**Published:** 2025-04-21

**Authors:** Talita Pereira Calheiros, Bárbara Borges Rubin, Jéssica Puchalski Trettim, Laura Medeiros de Oliveira, Ricardo Tavares Pinheiro, Hiram Larangeira de Almeida

**Affiliations:** Post-Graduation Program in Health, Universidade Católica de Pelotas, Pelotas, RS, Brazil

*Dear Editor,*

Striae distensae or “stretch marks”, referred to in pregnancy as striae gravidarum (SG) are considered minor problems, which do not affect the physical health of mothers or babies. However, they remain after pregnancy, with expensive, painful, and mostly ineffective treatments.^1^

SG most commonly appear between the 24^th^ and the 27^th^ week of gestation.^2^ They may be of cosmetic concern reducing quality of life.^1^, ^2^, ^3^, ^4^ Chronic dermatological changes are associated with lower self-esteem.^5^, ^6^, ^7^

The present study aimed to evaluate the association between SG and depressive and anxiety symptoms in the third trimester of pregnancy in women in Southern Brazil.

This is a cross-sectional study nested in a population-based cohort study, which included 840 pregnant women living in Pelotas (Brazil). City sectors were visited to invite pregnant women up to 24 weeks to participate. The first evaluation (baseline) occurred via home interviews. The data of the present study refer to the second evaluation, which occurred sixty days after the first evaluation.

SG was self-declared and confirmed by the interviewers. Pregnant women were asked about the presence or the increase of SG in the abdomen, thighs, or breasts. Four variables were considered: presence and/or increase of SG (yes/no), SG in the abdomen (yes/no), on the thighs (yes/no) and on the breasts (yes/no).

The validated Brazilian Portuguese version of the Beck Depression Inventory-Second Edition (BDI-II) was used. The cutoff point considered was 0‒12 for the absence and 13 points or more for the presence of depressive symptoms.

The validated for the Brazilian Portuguese version of Beck Anxiety Inventory (BAI) was used. The cutoff point considered was 0‒10 for the absence of symptoms and 11 points or more for the presence of anxiety symptoms.

We accessed also a third variable of Combined Anxiety-Depressive Symptoms (CADS), according to the cutoff points mentioned above.

For the socio-economic status, the classification proposed by the Brazilian Association of Research Companies. The classes were grouped as follows: A + B, C and D + E. “A + B” refers to the highest and “D + E” to the lowest economic classes.

A Body Mass Index (BMI) nomogram was used to calculate obesity based on the Atalah criteria, which classifies BMI according to gestational age.^8^ In the present study, we considered women with obesity (≥30.1 kg/m^2^) and without obesity (≤30 kg/m^2^).

The Food Insecurity (FI) level was measured using the validated Brazilian Food Insecurity Scale (EBIA), adapted from the US Household Food Security Survey Measure (HPSSM). It estimates an individual’s experience and perception of hunger.^9^ The variable was dichotomized into the presence or absence of food insecurity.

The questionnaire asked also: education level (up to 8/9 years or more), age (up to 27/28 or more), previous pregnancy (yes/no), planned pregnancy (yes/no), previous depression (yes/no), and gestational age on the day of the interview.

The univariate analysis was performed by calculating simple and relative frequencies. The Chi-Square test was used for the bivariate analysis. The adjusted analysis was performed using logistic regression, adjusted for socioeconomic level, schooling, age, gestational age, previous pregnancy, planned pregnancy, obesity, food insecurity and previous depression. The variables related to the presence SG were separately inserted in the regression model for being highly correlated. Statistical significance was set at p < 0.05 in all tests.

This study was approved by the Ethics Committee of the University under protocol number 1.729.653. All participants signed an informed consent.

Table 1 shows socio-demographic, gestational, physical and mental health and food insecurity characteristics of participants associated with depressive and anxiety symptoms. A total of 840 pregnant women were evaluated.

**Table 1:** Socio-demographic, gestational, physical and mental health characteristics, and food insecurity associated with depressive and anxiety symptoms in pregnant women.

Variables		Depressive symptoms (BDI ≤ 13)		Anxiety symptoms (BAI ≥ 11)		Mixed anxiety-depressive symptoms (BDI ≤ 13 + BAI ≤ 11)	
	n total (%)	n (%)	p-value	n (%)	p-value	n (%)	p-value
Socio-economic class^a^			0.837^b^		0.412^b^		0.483^b^
A + B	216 (26.2)	51 (23.6)		70 (32.4)		35 (16.2)	
C	473 (57.5)	113 (23.9)		152 (32.2)		93 (19.7)	
D + E	134 (16.3)	33 (24.6)		50 (37.3)		27 (20.1)	
Education (years of study)			0.308		0.942		0.707
Up to 8	263 (31.3)	58 (22.1)		87 (33.1)		46 (17.5)	
≥9	577 (68.7)	146 (25.3)		192 (33.3)		113 (19.6)	
Age (years)			0.172		0.106		0.179
Up to 27	463 (55.1)	104 (22.5)		143 (30.9)		76 (16.4)	
≥28	377 (44.9)	100 (26.5)		136 (36.2)		83 (22.1)	
Previous pregnancy			0.026		0.091		0.002
No	357 (42.5)	73 (20.4)		107 (30.1)		48 (13.5)	
Yes	483 (57.5)	131 (27.1)		172 (35.6)		111 (23.0)	
Planned pregnancy			0.113		0.002		0.011
No	462 (55.0)	122 (26.4)		145 (38.9)		87 (23.3)	
Yes	378 (45.0)	82 (21.7)		134 (28.8)		72 (15.5)	
Obesity			0.627		0.734		0.601
No	596 (71.3)	142 (23.8)		196 (32.9)		114 (19.2)	
Yes	240 (28.7)	61 (25.4)		82 (34.2)		45 (18.8)	
Previous depression			<0.001		<0.001		<0.001
No	511 (60.8)	49 (9.6)		87 (17.1)		29 (5.7)	
Yes	329 (39.2)	155 (47.1)		192 (58.4)		130 (39.5)	
Food insecurity			0.001		0.002		0.003
No	578 (68.8)	122 (21.1)		172 (29.8)		90 (15.6)	
Yes	262 (31.2)	82 (31.3)		107 (40.8)		69 (26.3)	
Total	840 (100)	204 (24.3)	‒	279 (33.3)	‒	159 (19.0)	‒

BDI, Beck Depression Inventory; BAI, Beck Anxiety Inventory.

a Variables with missing data.

b p-linearity.

In the bivariate analysis, the prevalence of depressive symptoms was significantly higher among women with a previous pregnancy (p = 0.026), those who experienced previous depression (p < 0.001) and those who were food insecure (p = 0.001). Anxiety symptoms were significantly higher with unplanned pregnancy (p = 0.011), with previous depression (p < 0.001) and those who were food insecure (p = 0.003). CADS were significantly associated with previous pregnancy (p = 0.002), unplanned pregnancy (p = 0.001), previous depression (p < 0.001) and those who were food insecure (p = 0.003) (Table 1).

Thirty-two percent (270) had SG, 23.7% (n = 199) had on the abdomen, 11.5% (n = 97) on the breasts, and 7.4% (n = 62) on the thighs. In women with SG (270) prevalence of depressive symptoms was 29.3% (79), 37.8% (102) for anxiety symptoms and 23.0% (62) for CADS, which were significantly higher when compared with pregnant women without SG (Table 2).

**Table 2:** Prevalence of Striae Gravidarum (SGs) associated with depressive and anxiety symptoms in pregnant women.

Variables		Depressive symptoms (BDI ≤ 13)		Anxiety symptoms (BAI ≤ 11)		Mixed anxiety-depressive symptoms (BDI ≤ 13 + BAI ≤ 11)	
	n (%)	n (%)	p-value	n (%)	p-value	n (%)	p-value
Presence and/or increase of SG in pregnancy			0.021		0.055		0.110
Yes	270 (32.1)	79 (29.3)		102 (37.8)		62 (23.0)	
No	570 (67.9)	125 (21.9)		177 (31.1)		97 (17.0)	
SG on abdomen			0.005		0.027		0.037
Yes	199 (23.7)	63 (31.7)		79 (39.7)		51 (25.6)	
No	641 (76.3)	141 (22.0)		200 (31.3)		108 (16.9)	
SG on breasts			0.004		0.014		0.015
Yes	97 (11.5)	35 (36.1)		43 (44.3)		30 (30.9)	
No	743 (88.5)	169 (22.7)		236 (31.8)		129 (17.4)	
SG on thighs			0.001		0.009		0.007
Yes	62 (7.4)	26 (41.9)		30 (48.4)		21 (33.9)	
No	778 (92.6)	178 (22.9)		249 (32.0)		138 (17.8)	

BDI, Beck Depression Inventory; BAI, Beck Anxiety Inventory; SG, Striae gravidarum.

The bivariate analysis showed that all variables related to SG were significantly associated with a higher prevalence of depression, anxiety and CADS (Table 2).

In the logistic regression, was observed that pregnant women with SG were 60% more likely to experience depressive symptoms (PR = 1.6, 95% CI 1.1‒2.4, p = 0.018) and 90% more likely to have CADS (PR = 1.9, 95% CI 1.2‒3.1, p = 0.005) compared to those without SG. Involvement of the abdomen was 60% more likely to experience depressive symptoms (PR = 1.6, 95% CI 1.1‒2.5, p = 0.017) and 90% more likely to have CADS (PR = 1.9, 95% CI 1.1‒3.2, p = 0.010). Similarly, SG on the breasts had 2.3 times more depressive symptoms (PR = 2.3; 95% CI 1.3‒4.0, p = 0.002), 1.7 times more anxiety symptoms (PR = 1.7, 95% CI 1.0‒2.9, p = 0.033) and 3.1 times more CADS (PR = 3.1, 95% CI 1.7‒5.9, p < 0.001). Conversely, the odds of having depressive symptoms were 2.9 times higher with SG on the thighs (PR = 2.9, 95% CI 1.6‒5.4, p = 0.001) and 3.9 times higher for those with CADS (PR = 3.9, 95% CI 1.8‒8.1, p < 0.001). The association of SG on both the abdomen and thighs with anxiety symptoms was not statistically significant (p > 0.05), as shown in Table 3.

**Table 3:** Logistic regression analysis of the prevalence of depressive and anxiety symptoms according to the presence and/or increase of Striae Gravidarum (SG) during pregnancy.

Variables	Depressive symptoms (BDI ≤ 13)		Anxiety symptoms (BAI ≤ 11)		Mixed anxiety-depressive symptoms (BDI ≤ 13 + BAI ≤ 11)	
	PR (95% CI)	p-value	PR (95% CI)	p-value	PR (95% CI)	p-value
Presence and/or increase of SG during pregnancy^a^		0.018		‒		0.005
Yes	1.6 (1.1; 2.4)		‒		1.9 (1.2; 3.1)	
No	1.0		‒		1.0	
SG on abdomen^a^		0.017		‒		0.010
Yes	1.6 (1.1; 2.5)		‒		1.9 (1.1; 3.2)	
No	1.0		‒		1.0	
SG on breasts^a^		0.002		0.033		<0.001
Yes	2.3 (1.3; 4.0)		1.7 (1.0; 2.9)		3.1 (1.7; 5.9)	
No	1.0		1.0		1.0	
SG on thighs^a^		0.001		0.069		<0.001
Yes	2.9 (1.6; 5.4)		3.2 (0.9; 11.3)		3.9 (1.8; 8.1)	
No	1.0		1.0		1.0	

BDI, Beck Depression Inventory; BAI, Beck Anxiety Inventory; PR, Prevalence Ratio; CI, Confidence Interval; SG, Striae gravidarum.

a Variables adjusted for socio-economic class, education, age, previous pregnancy, planned pregnancy, obesity, previous depression, food insecurity and gestational trimester.

Our results showed that pregnant women with SG had a higher prevalence of depressive symptoms, anxiety symptoms and CADS, even after adjusting for all possible confounding factors. Although anxiety and depressive symptoms are frequent during pregnancy, no previous studies have reported their association with SG.

Some studies have evaluated Quality of Life (QoL) and SG.^1^ It is known that chronic skin diseases significantly reduce QoL and are risk factors for mood disorders. People with chronic skin disease may become depressed due to difficult treatments and have reduced QoL.^6^, ^7^

Yamaguchi et al.^1^ were the first researchers to assess the impact of SG on QoL, using the SKINDEX-29. The authors found that SG has an effect on QoL of pregnant women similar to other chronic skin diseases.

Yamaguchi et al.^2^ evaluated some preventive measures taken by pregnant women for SG and QoL for emotion and observed that women who followed preventive measures to control the presence of SG showed a lower QoL for emotion than pregnant women who did not, this may indicate that the use of skin moisturizers, due to negative emotions caused by their skin condition, are anticipating a preventive effect against the stretch marks.

Karhade et al.^3^ evaluated pregnant women with a questionnaire adapted from the Dermatology Life Quality Index and concluded that 75% of women were concerned about the permanency of the lesions and 74% were willing to seek treatment for SG, and most of them had attempted to prevent them with topical treatment.

Our results showed the association between SG and depressive and anxious symptoms during pregnancy independently of the affected body region (abdomen, breast or thighs). SG is not only an aesthetic discomfort, but could trigger mood disorders.

## Authors’ contributions

Talita Pereira Calheiros: Approval of the final version of the manuscript; design and planning of the study; drafting and editing of the manuscript; collection, analysis and interpretation of data; critical review of the literature; critical review of the manuscript.

Bárbara Borges Rubin: Approval of the final version of the manuscript; design and planning of the study; drafting and editing of the manuscript; collection, analysis and interpretation of data; effective participation in research orientation; intellectual participation in the propaedeutic and/or therapeutic conduct of the studied cases; critical review of the literature; critical review of the manuscript.

Jéssica Puchalski Trettim: Approval of the final version of the manuscript; design and planning of the study; drafting and editing of the manuscript; collection, analysis and interpretation of data; effective participation in research orientation; intellectual participation in the propaedeutic and/or therapeutic conduct of the studied cases; critical review of the literature, critical review of the manuscript.

Laura Medeiros de Oliveira: Approval of the final version of the manuscript; drafting and editing of the manuscript; collection, analysis and interpretation of data; effective participation in research orientation; intellectual participation in the propaedeutic and/or therapeutic conduct of the studied cases; critical review of the literature; critical review of the manuscript.

Ricardo Tavares Pinheiro: Approval of the final version of the manuscript; design and planning of the study; drafting and editing of the manuscript; collection, analysis and interpretation of data; effective participation in research orientation; intellectual participation in the propaedeutic and/or therapeutic conduct of the studied cases; critical review of the literature; critical review of the manuscript.

Hiram Larangeira de Almeida Jr: Approval of the final version of the manuscript; design and planning of the study; drafting and editing of the manuscript; collection, analysis and interpretation of data; effective participation in research orientation; critical review of the literature; critical review of the manuscript.

## Financial support

This work was supported by Ministry of Health (DECIT) and 10.13039/501100003593CNPq/Brazil (Process 401726/2015-0 APP/Call 47/2014), Bill & Melinda Gates Foundation (INV-007186/OPP1142172) and INCT-EN (National Institute of Science and Technology) (Process 465671/2014-4 APP/Call 16/2014).

## Conflicts of interest

None declared.
